# Bis(2,4,6-tri­amino­pyrimidin-1-ium) sulfate penta­hydrate

**DOI:** 10.1107/S1600536813019223

**Published:** 2013-07-17

**Authors:** Ruthairat Nimthong, Siva Chamchong, Chaveng Pakawatchai, Jedsada Mokhagul, Yupa Wattanakanjana

**Affiliations:** a Department of Chemistry, Faculty of Science, Prince of Songkla University, Hat Yai, Songkhla 90112, Thailand

## Abstract

The asymmetric unit of the title salt, 2C_4_H_8_N_5_^+^·SO_4_^2−^·5H_2_O, contains four 2,4,6-tri­amino­pyrimidinium (TAPH^+^) cations, two sulfate anions and ten lattice water mol­ecules. Each two of the four TAPH^+^ cations form dimers *via* N—H⋯N hydrogen bonds between the amino groups and the unprotonated pyrimidine N atoms [graph-set motif *R*_2_^2^(8)]. The (TAPH^+^)_2_ dimers, in turn, form slightly offset infinite π–π stacks parallel to [010], with centroid–centroid distances between pyrimidine rings of 3.5128 (15) and 3.6288 (16) Å. Other amino H atoms, as well as the pyrimidinium N—H groups, are hydrogen-bonded to sulfate and lattice water O atoms. The SO_4_^2−^ anions and water mol­ecules are inter­connected with each other *via* O—H⋯O hydrogen bonds. The combination of hydrogen-bonding inter­actions and π–π stacking leads to the formation of a three-dimensional network with alternating columns of TAPH^+^ cations and channels filled with sulfate anions and water mol­ecules. One of the sulfate anions shows a minor disorder by a *ca* 37° rotation around one of the S—O bonds [occupancy ratio of the two sets of sites 0.927 (3):0.073 (3)]. One water mol­ecule is disordered over two mutually exclusive positions with an occupancy ratio of 0.64 (7):0.36 (7).

## Related literature

For background to melamine, see: Wei & Liu (2012 ▶); Dobson *et al.* (2008 ▶); Whitesides *et al.* (1991 ▶). For pyrimidine–metal complexes, see: Zamora *et al.* (1997 ▶); Louloudi *et al.* (1997 ▶); Jolibois *et al.* (1998 ▶); Katritzky *et al.* (1984 ▶). For carbon protonation of pyrimidines, see: Demeter & Wéber (2004 ▶); Németh *et al.* (2006 ▶). For related structures, see: Hemamalini *et al.* (2005 ▶); Krygowski *et al.* (2005 ▶). For graph-set analysis, see: Etter *et al.* (1990 ▶).
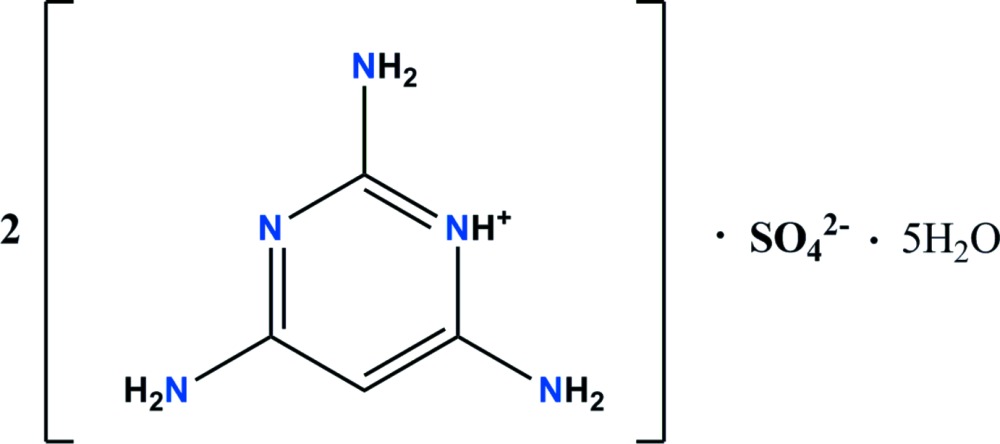


## Experimental

### 

#### Crystal data

2C_4_H_8_N_5_^+^·SO_4_^2−^·5H_2_O
*M* *_r_* = 438.45
Triclinic, 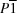

*a* = 10.6571 (7) Å
*b* = 13.2482 (9) Å
*c* = 15.0132 (10) Å
α = 100.843 (2)°
β = 110.596 (2)°
γ = 92.096 (2)°
*V* = 1936.6 (2) Å^3^
*Z* = 4
Mo *K*α radiation
μ = 0.23 mm^−1^
*T* = 293 K
0.22 × 0.11 × 0.03 mm

#### Data collection

Bruker APEX CCD diffractometer
Absorption correction: multi-scan (*SADABS*; Bruker, 2003 ▶) *T*_min_ = 0.762, *T*_max_ = 1
22960 measured reflections
9354 independent reflections
5782 reflections with *I* > 2σ(*I*)
*R*_int_ = 0.052

#### Refinement

*R*[*F* ^2^ > 2σ(*F* ^2^)] = 0.077
*wR*(*F* ^2^) = 0.170
*S* = 1.08
9354 reflections
601 parameters
40 restraints
H atoms treated by a mixture of independent and constrained refinement
Δρ_max_ = 0.37 e Å^−3^
Δρ_min_ = −0.28 e Å^−3^

### 

Data collection: *SMART* (Bruker, 2003 ▶); cell refinement: *SAINT* (Bruker, 2003 ▶); data reduction: *SAINT*; program(s) used to solve structure: *SHELXS2013* (Sheldrick, 2008 ▶); program(s) used to refine structure: *SHELXL2013* and *SHELXLE* (Hübschle *et al.*, 2011 ▶); molecular graphics: *Mercury* (Macrae *et al.*, 2008 ▶); software used to prepare material for publication: *SHELXL2013* and *publCIF* (Westrip, 2010 ▶).

## Supplementary Material

Crystal structure: contains datablock(s) I, New_Global_Publ_Block. DOI: 10.1107/S1600536813019223/wm2755sup1.cif

Structure factors: contains datablock(s) I. DOI: 10.1107/S1600536813019223/wm2755Isup2.hkl

Supplementary material file. DOI: 10.1107/S1600536813019223/wm2755Isup3.cml

Additional supplementary materials:  crystallographic information; 3D view; checkCIF report

## Figures and Tables

**Table 1:** Hydrogen-bond geometry (Å, °)

*D*—H⋯*A*	*D*—H	H⋯*A*	*D*⋯*A*	*D*—H⋯*A*
N4*B*—H4*BA*⋯O17^i^	0.86	2.15	2.967 (4)	159
N4*B*—H4*BB*⋯O8	0.86	2.07	2.905 (4)	162
N4*B*—H4*BB*⋯O5*B*	0.86	2.38	3.19 (4)	157
N4*A*—H4*AA*⋯N3*C* ^ii^	0.86	2.16	3.017 (4)	172
N4*A*—H4*AB*⋯O14	0.86	2.56	3.262 (4)	139
N5*B*—H5*BA*⋯N3*D* ^ii^	0.86	2.22	3.074 (4)	174
N5*B*—H5*BB*⋯O7^iii^	0.86	2.20	3.040 (4)	166
N5*B*—H5*BB*⋯O7*B* ^iii^	0.86	2.10	2.93 (3)	163
N5*A*—H5*AA*⋯O1^i^	0.86	2.25	2.993 (3)	145
N5*A*—H5*AB*⋯O12	0.86	2.21	3.066 (4)	174
N4*D*—H4*DA*⋯N3*B* ^iv^	0.86	2.16	3.013 (4)	176
N4*D*—H4*DB*⋯O17^v^	0.86	2.57	3.248 (4)	137
N4*C*—H4*CA*⋯O12^vi^	0.86	2.14	2.953 (4)	158
N4*C*—H4*CB*⋯O1	0.86	2.16	2.983 (3)	160
N1*D*—H1*D*⋯O9^v^	0.86	1.97	2.826 (4)	170
N1*C*—H1*CB*⋯O4	0.86	1.86	2.709 (3)	172
N1*B*—H1*B*⋯O6	0.86	1.92	2.757 (6)	166
N1*B*—H1*B*⋯O6*B*	0.86	1.91	2.75 (7)	164
N1*A*—H1*A*⋯O14	0.86	1.95	2.793 (4)	166
N5*D*—H5*DA*⋯O8^vi^	0.86	2.31	3.046 (4)	143
N5*D*—H5*DA*⋯O5*B* ^vi^	0.86	2.23	2.99 (5)	147
N5*D*—H5*DB*⋯O17	0.86	2.34	3.193 (4)	173
N5*C*—H5*CA*⋯N3*A* ^iv^	0.86	2.14	2.990 (4)	172
N5*C*—H5*CB*⋯O3^v^	0.86	2.06	2.905 (3)	169
N6*A*—H6*AA*⋯O13	0.86	2.14	2.981 (4)	166
N6*A*—H6*AB*⋯O10	0.86	2.02	2.855 (4)	164
O9—H9*C*⋯O7^vii^	0.80 (2)	1.98 (2)	2.770 (4)	171 (4)
O9—H9*C*⋯O8*B* ^vii^	0.80 (2)	2.24 (5)	2.84 (3)	133 (3)
N6*D*—H6*DA*⋯O16	0.86	2.08	2.937 (4)	179
N6*D*—H6*DB*⋯O15	0.86	2.16	3.005 (4)	166
N6*C*—H6*CA*⋯O13	0.86	2.14	2.970 (4)	162
N6*B*—H6*BA*⋯O6	0.86	2.60	3.284 (5)	137
N6*B*—H6*BA*⋯O6*B*	0.86	2.64	3.31 (4)	135
N6*B*—H6*BA*⋯O15	0.86	2.63	3.427 (4)	154
N6*B*—H6*BB*⋯O16	0.86	2.10	2.923 (4)	159
O9—H9*D*⋯O2	0.83 (2)	1.93 (2)	2.759 (4)	173 (4)
O10—H10*C*⋯O4	0.83 (2)	1.96 (2)	2.780 (4)	172 (6)
O10—H10*D*⋯O14	0.81 (2)	2.51 (6)	3.026 (5)	123 (6)
O10—H10*D*⋯O18^iii^	0.81 (2)	2.09 (5)	2.82 (3)	151 (6)
O11—H11*C*⋯O18^iii^	0.86 (2)	2.40 (7)	2.97 (4)	124 (5)
O11—H11*C*⋯O18*B* ^iii^	0.86 (2)	2.12 (4)	2.826 (18)	139 (5)
O11—H11*D*⋯O3	0.85 (2)	1.90 (2)	2.745 (4)	171 (5)
O12—H12*A*⋯O5	0.82 (2)	1.98 (2)	2.795 (4)	172 (5)
O12—H12*A*⋯O5*B*	0.82 (2)	2.05 (4)	2.81 (3)	156 (5)
O12—H12*B*⋯O3^viii^	0.82 (2)	2.06 (2)	2.856 (4)	165 (5)
O13—H13*C*⋯O6	0.82 (2)	2.15 (2)	2.956 (6)	167 (5)
O13—H13*C*⋯O6*B*	0.82 (2)	2.06 (7)	2.87 (6)	167 (5)
O13—H13*D*⋯O11^viii^	0.81 (2)	2.26 (2)	3.063 (5)	175 (5)
O14—H14*C*⋯O5^iii^	0.81 (2)	2.06 (3)	2.792 (4)	152 (5)
O14—H14*C*⋯O7*B* ^iii^	0.81 (2)	2.21 (4)	2.98 (4)	159 (5)
O14—H14*D*⋯O11^ix^	0.81 (2)	1.95 (2)	2.761 (4)	172 (5)
O15—H15*C*⋯O9^v^	0.84 (2)	2.10 (3)	2.894 (4)	158 (5)
O15—H15*D*⋯O6	0.83 (2)	2.02 (3)	2.808 (7)	158 (5)
O15—H15*D*⋯O6*B*	0.83 (2)	2.11 (8)	2.90 (8)	159 (5)
O16—H16*C*⋯O15^vii^	0.81 (2)	2.05 (2)	2.855 (5)	171 (5)
O16—H16*D*⋯O2	0.82 (2)	2.07 (2)	2.882 (4)	175 (5)
O17—H17*A*⋯O1	0.82 (2)	2.09 (2)	2.878 (4)	162 (4)
O17—H17*B*⋯O8^vii^	0.81 (2)	2.07 (2)	2.837 (4)	160 (5)
O17—H17*B*⋯O8*B* ^vii^	0.81 (2)	1.96 (4)	2.74 (3)	163 (5)
O18—H18*C*⋯O7	0.84 (2)	2.04 (11)	2.78 (2)	147 (18)
O18—H18*D*⋯O2^v^	0.84 (2)	2.21 (2)	2.92 (2)	143 (5)
O18*B*—H18*E*⋯O7*B*	0.84 (2)	1.83 (11)	2.49 (4)	134 (13)
O18*B*—H18*F*⋯O2^v^	0.83 (2)	2.10 (2)	2.857 (14)	152 (6)

Symmetry codes: (i) 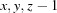; (ii) 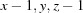; (iii) 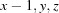; (iv) 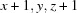; (v) 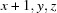; (vi) 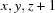; (vii) 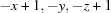; (viii) 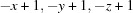; (ix) 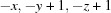.

**Fig. 1.**
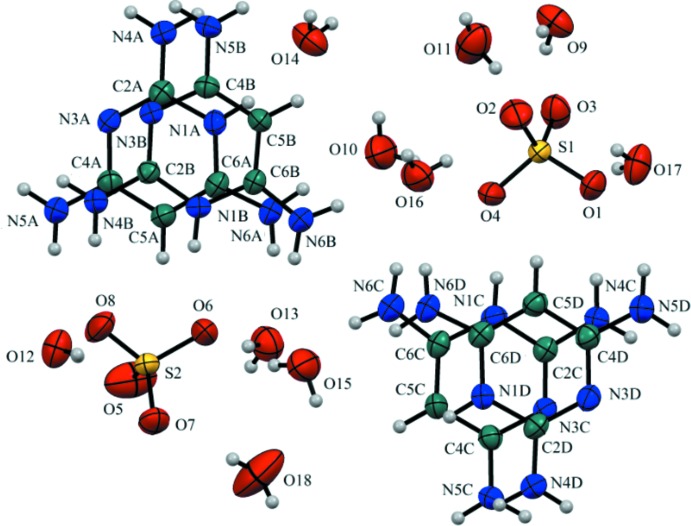
The asymmetric unit of the title compound. Displacement ellipsoids are drawn at the 50% probability level. The minor disordered moieties are omitted for clarity.

**Fig. 2.**
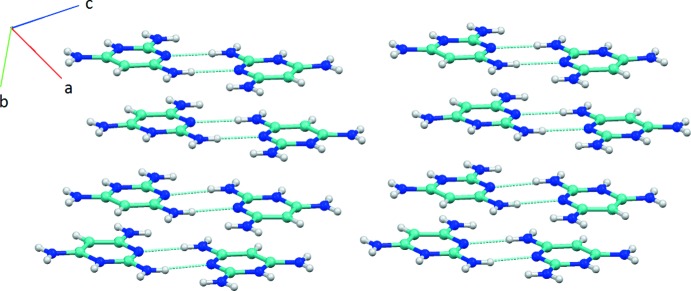
Part of the crystal structure of (I) showing intermolecular N—H···N hydrogen bonds (blue dashed lines) between TAPH+ cations and π–π-stacks parallel to [010].

**Fig. 3.**
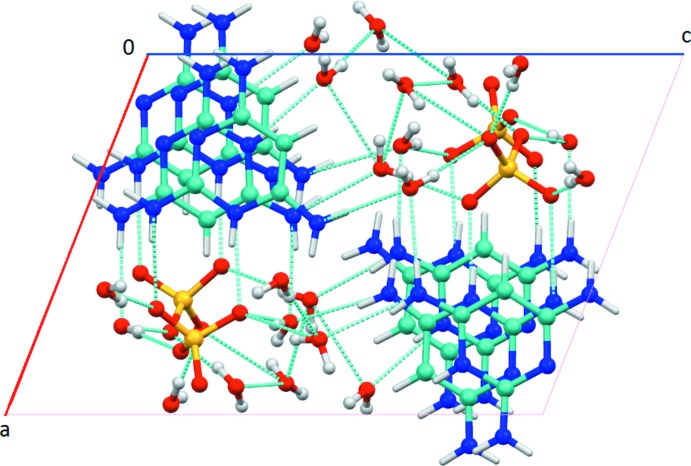
The packing structure of the title complex viewed down [010].

**Fig. 4.**
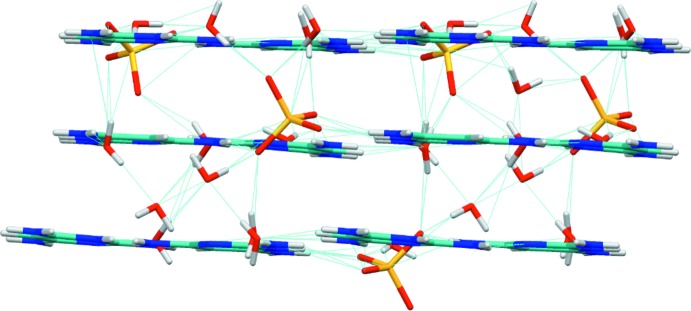
Part of the crystal structure showing formation of a tightly interconnected three-dimensional network with alternating columns of TAPH cations and channels filled with sulfate anions and water molecules.

## supplementary crystallographic information

### Comment

Pyrimidine and its derivatives are a class of heteroaromatic compounds with exceptional importance for biology, pharmacology and live sciences. An immensely large number of naturally occurring compounds are based on the pyrimidine skeleton, including three of the four DNA and RNA nucleobases, cytosine, thymine, and uracil. The pyrimidine ring, while less basic than equivalent pyridines, can nonetheless be protonated with relative ease, at either one of the two nitrogen ring atoms (double protonation is hampered by near complete loss of basicity upon the first protonation), or at the ring carbon atom (Demeter & Wéber, 2004; Németh *et al.*, 2006). It can also act as a Lewis base in metal complex formation (Zamora *et al.*, 1997; Louloudi *et al.*, 1997; Jolibois *et al.*, 1998; Katritzky *et al.*, 1984), or as an acceptor for strong hydrogen bonding interactions. These three properties of pyrimidines are the key aspects to the wide range of their biological and pharmacological functionalities, both natural as well as synthetic.

Amino-substituted pyrimidines are of interest due to their similarity to the nucleic acids cytosine, adenine and guanine. Multi-amino substituted derivatives have recently attracted intense attention due to their similarity with melamine, which had been added to dairy products and other food to give a false appearance of a higher protein level. Exposure to high levels of melamine in food can however lead to melamine-induced kidney failure, and adulterated food had been the cause for several severe outbreaks of nephrolithiasis in pets ("2007 pet food recall") and humans ("2008 Chinese milk scandal" with more than 50,000 infant hospitalizations and six deaths) (Wei & Liu, 2012). The cause for the melamine-induced kidney stone formation was found to be a highly insoluble co-crystalline precipitate of melamine and uric acid, a hydrolysis product of melanine itself (Dobson *et al.*, 2008). The insolubility of the melamine-uric acid co-crystal can be largely traced back to dense π–π stacking interactions and the formation of a network of strong N—H···O and N—H···N hydrogen bonds (Whitesides *et al.*, 1991). The propensity of melamine and its derivatives to form tightly hydrogen-bonded insoluble networks is thus of great interest.

One such multi-amino substituted pyrimidine derivative is 2,4,6-triaminopyrimidine, which finds, for example, use as an internal standard in testing for melamine in food. In this communication we present the structure of the sulfate salt of 2,4,6-triaminopyrimidine, in the form of its pentahydrate, (C_4_H_8_N_5_)_2_^+^SO_4_^2-^^.^5H_2_O, (I).

The asymmetric unit of compound (I) consists of four mono-protonated 2,4,6-triaminopyrimidine cations (TAPH^+^), two sulfate anions and ten water molecules (Fig. 1). All four TAPH^+^ cations are protonated at one of the pyrimidine ring nitrogen atoms (N1 atoms in molecules A, B, C and D). As it is common for pyridyl derivatives, the bond angles at the protonated nitrogen are slightly larger than those at the unprotonated nitrogen atoms (Krygowski *et al.*, 2005). The C—N—C angles range between 120.7 (3)–121.2 (3)° at protonated N1 in the four independent cations. At the unprotonated nitrogen atom the equivalent angles are substantially smaller, with values between 116.7 (3) and 117.3 (3)°. Similar trends are observed for other amino substituted pyrimidine derivative salts such as the hydrogen sulfate salt of 2-amino-4,6-dimethylpyrimidinium, C_6_H_10_N_3_^+^^.^HSO_4_^-^, with an angle at the protonated nitrogen of 122.3 (1)° and of 117.6 (1)° at the unprotonated nitrogen atom, respectively (Hemamalini *et al.*, 2005). S—O bonds lengths of the tetrahedral sulfate anions are in the range from 1.449 (4) Å to 1.471 (4) Å, indicating delocalized S≐O bonds rather than distinct single and double bonds. The amino groups, which are not protonated, are *sp*^2^ hybridized and the NH_2_ groups are coplanar with the pyrimidinium rings.

The packing of the molecules is dominated by a mixture of π–π-stacking and hydrogen bonding interactions. The primary packing motif formed by the TAPH^+^ cations are hydrogen-bonded dimers. Pairs of N—H···N hydrogen bonds between the amino NH_2_ groups and the unprotonated pyrimidine nitrogen atoms of each two of the four TAPH^+^ cations form dimers (graph set motif *R*_2_^2^(8); Etter *et al.*, 1990). The dimers, formed between cations A and C and B and D, respectively, have local pseudoinversion symmetry. The thus formed (TAPH^+^)_2_ dimers are in turn forming slightly offset π–π- stacks that stretch parallel to [010] (Fig. 2). Centroid–centroid distances between individual pyrimidine rings are between 3.5128 (15) and 3.6288 (16) Å. Interplanar distances are, due to the offset between the stacked dimers, substantially shorter and range between 3.2456 (11) and 3.2847 (11) Å.

The thus formed columns of TAPH^+^ cations make up about half of the unit cell volume (Fig. 3). The remainder of the volume is taken up by the sulfate anions and lattice water molecules. The H atoms of amino groups that are not engaged in N—H···N hydrogen bonds as well as the pyrimidine N—H groups of the TAPH^+^ cations are hydrogen-bonded through N—H···O hydrogen bonds to oxygen atoms of sulfate anions and water molecules. The SO_4_^2-^ tetrahedra and H_2_O molecules are in turn interconnected with each other *via* O—H···O hydrogen bonds.

The combination of π-stacking interactions and hydrogen bonding leads to the formation of a tightly interconnected three-dimensional network with alternating columns of TAPH^+^ cations and channels filled with sulfate anions and water molecules (Fig. 4).

### Experimental

Crystals of the title compound were isolated as an unintended by-product of the reaction of 2,4,6-triaminopyrimidine with phenylisothiocyanate when attempting to synthesize diaminopyrimidinyl-phenylthiourea. 2,4,6-Triaminopyrimidine, TAP, (0.36 g, 2.88 mmol) was dissolved in 40 cm^3^ of ethanol at 333 K. Phenylisothiocyanate (0.35 ml, 2.93 mmol) was added and the mixture was stirred for 1 h. The resulting clear solution was filtered and left to evaporate at room temperature. The crystalline material that formed upon standing for several days was filtered off and dried *in vacuo* (yield 0.4 g). A crystal was selected from the material and subjected to single-crystal structure analysis. No attempts were made to further analyze the remainder of the material.

### Refinement

H atoms bonded to C and N atoms were constrained to ride on their parent atoms with C—H bond lengths of 0.93 Å for aryl C—H and N—H bond lengths of 0.86 Å with *U*_iso_(H) = 1.2*U*_eq_(C and N). All H atoms bonded to O atoms were located in a difference Fourier map and were refined isotropically. *U*_iso_ values of water hydrogen atoms were constrained to 1.5 times the *U*_eq_ value of their oxygen carrier atom. The structure exhibits two independent types of disorder. One of the sulfate anions (S2) shows minor disorder by a *ca* 37° rotation around one of the S—O bonds with a refined occupancy ratio of 0.927 (3):0.073 (3). One of the water molecules (O18) shows disorder over two mutually exclusive positions with refined occupancies of 0.64 (7):0.36 (7). For the sulfate ion, minor moiety atoms were constrained to have the same anisotropic displacement parameters, ADP, as their major moiety counterparts. The ADPs of the oxygen atoms of the disordered water molecule were restrained to have similar *U_ij_* components (e.s.d. = 0.04 Å^2^). All O—H bond lengths in water molecules were restrained to a target value of 0.82 (2) Å. For the H atoms of the less prevalent moiety of the disordered water molecule, O···H distances were restrained based on hydrogen bonding considerations (2.10 (2) Å for O2^i^···H18F and 2.20 (2) Å for O2 ^i^···H18D (symmetry operator (i): 1 + *x*, +*y*, +*z*).

### Crystal data

**Table:**

2C_4_H_8_N_5_^+^·SO_4_^2^^−^·5H_2_O	*Z* = 4
*M**_r_* = 438.45	*F*(000) = 928
Triclinic, *P*1	*D*_x_ = 1.504 Mg m^−^^3^
Hall symbol: -P 1	Mo *K*α radiation, λ = 0.71073 Å
*a* = 10.6571 (7) Å	Cell parameters from 1991 reflections
*b* = 13.2482 (9) Å	θ = 2.9–22.1°
*c* = 15.0132 (10) Å	µ = 0.23 mm^−^^1^
α = 100.843 (2)°	*T* = 293 K
β = 110.596 (2)°	Block, yellow
γ = 92.096 (2)°	0.22 × 0.11 × 0.03 mm
*V* = 1936.6 (2) Å^3^	

### Data collection

**Table:**

Bruker APEX CCD diffractometer	5782 reflections with *I* > 2σ(*I*)
Radiation source: sealed tube	*R*_int_ = 0.052
Phi and ω scans	θ_max_ = 28.1°, θ_min_ = 1.5°
Absorption correction: multi-scan (*SADABS*; Bruker, 2003)	*h* = −14→14
*T*_min_ = 0.762, *T*_max_ = 1	*k* = −17→17
22960 measured reflections	*l* = −19→18
9354 independent reflections	

### Refinement

**Table:**

Refinement on *F*^2^	Primary atom site location: structure-invariant direct methods
Least-squares matrix: full	Secondary atom site location: difference Fourier map
*R*[*F*^2^ > 2σ(*F*^2^)] = 0.077	Hydrogen site location: inferred from neighbouring sites
*wR*(*F*^2^) = 0.170	H atoms treated by a mixture of independent and constrained refinement
*S* = 1.08	*w* = 1/[σ^2^(*F*_o_^2^) + (0.0614*P*)^2^ + 0.4094*P*] where *P* = (*F*_o_^2^ + 2*F*_c_^2^)/3
9354 reflections	(Δ/σ)_max_ < 0.001
601 parameters	Δρ_max_ = 0.37 e Å^−^^3^
40 restraints	Δρ_min_ = −0.28 e Å^−^^3^

### Special details

**Table:**

Geometry. All e.s.d.'s (except the e.s.d. in the dihedral angle between two l.s. planes) are estimated using the full covariance matrix. The cell e.s.d.'s are taken into account individually in the estimation of e.s.d.'s in distances, angles and torsion angles; correlations between e.s.d.'s in cell parameters are only used when they are defined by crystal symmetry. An approximate (isotropic) treatment of cell e.s.d.'s is used for estimating e.s.d.'s involving l.s. planes.

### Fractional atomic coordinates and isotropic or equivalent isotropic displacement parameters (Å^2^)

**Table:**

	*x*	*y*	*z*	*U*_iso_*/*U*_eq_	Occ. (<1)
S1	0.31694 (8)	0.31861 (6)	0.75524 (5)	0.0316 (2)	
O1	0.3919 (2)	0.3087 (2)	0.85473 (16)	0.0523 (7)	
O2	0.2346 (2)	0.22044 (18)	0.69832 (18)	0.0550 (7)	
C2C	0.7573 (3)	0.3707 (2)	0.8800 (2)	0.0293 (7)	
O3	0.2280 (3)	0.39993 (18)	0.75571 (17)	0.0530 (7)	
O4	0.4109 (2)	0.3429 (2)	0.70841 (16)	0.0488 (6)	
C4C	0.9531 (3)	0.3724 (2)	0.8494 (2)	0.0315 (7)	
S2	0.7903 (3)	0.1546 (2)	0.2933 (2)	0.0342 (2)	0.927 (3)
O5	0.8248 (4)	0.2552 (3)	0.2780 (3)	0.0894 (12)	0.927 (3)
O6	0.7184 (6)	0.1688 (5)	0.3602 (4)	0.0617 (9)	0.927 (3)
O7	0.9162 (3)	0.1100 (2)	0.33715 (18)	0.0483 (7)	0.927 (3)
O8	0.7067 (3)	0.0885 (2)	0.20102 (19)	0.0627 (9)	0.927 (3)
S2B	0.790 (3)	0.150 (2)	0.293 (3)	0.0342 (2)	0.073 (3)
O5B	0.743 (4)	0.205 (3)	0.214 (3)	0.0894 (12)	0.073 (3)
O6B	0.717 (6)	0.177 (6)	0.358 (5)	0.0617 (9)	0.073 (3)
O7B	0.935 (3)	0.181 (3)	0.345 (2)	0.0483 (7)	0.073 (3)
O8B	0.765 (3)	0.040 (2)	0.253 (2)	0.0627 (9)	0.073 (3)
N3B	0.2310 (2)	0.13336 (18)	0.15229 (17)	0.0290 (6)	
C2B	0.3628 (3)	0.1343 (2)	0.1830 (2)	0.0280 (6)	
C2A	0.1864 (3)	0.3781 (2)	0.1557 (2)	0.0306 (7)	
N3A	0.2487 (2)	0.37711 (19)	0.09404 (17)	0.0327 (6)	
C2D	0.9274 (3)	0.1235 (2)	0.9110 (2)	0.0324 (7)	
N4B	0.4275 (3)	0.1298 (2)	0.12153 (19)	0.0406 (7)	
H4BA	0.3832	0.1262	0.0606	0.049*	
H4BB	0.5138	0.1305	0.1426	0.049*	
C4B	0.1731 (3)	0.1369 (2)	0.2198 (2)	0.0290 (7)	
N4A	0.0528 (3)	0.3711 (2)	0.12353 (19)	0.0405 (7)	
H4AA	0.0074	0.3660	0.0625	0.049*	
H4AB	0.0116	0.3717	0.1636	0.049*	
N3D	0.8648 (3)	0.12722 (19)	0.97309 (18)	0.0344 (6)	
N3C	0.8900 (2)	0.37463 (18)	0.91433 (17)	0.0308 (6)	
C4A	0.3854 (3)	0.3852 (2)	0.1309 (2)	0.0335 (7)	
N5B	0.0394 (3)	0.1343 (2)	0.1869 (2)	0.0426 (7)	
H5BA	−0.0049	0.1305	0.1260	0.051*	
H5BB	−0.0026	0.1363	0.2266	0.051*	
C5B	0.2473 (3)	0.1422 (2)	0.3185 (2)	0.0324 (7)	
H5B	0.2047	0.1442	0.3632	0.039*	
N5A	0.4473 (3)	0.3823 (2)	0.0675 (2)	0.0488 (8)	
H5AA	0.4007	0.3755	0.0065	0.059*	
H5AB	0.5339	0.3871	0.0875	0.059*	
C4D	0.7289 (3)	0.1243 (2)	0.9359 (2)	0.0334 (7)	
N4D	1.0610 (3)	0.1265 (2)	0.9424 (2)	0.0458 (7)	
H4DA	1.1066	0.1308	1.0033	0.055*	
H4DB	1.1018	0.1241	0.9019	0.055*	
N4C	0.6882 (3)	0.3737 (2)	0.93852 (19)	0.0380 (6)	
H4CA	0.7299	0.3783	0.9999	0.046*	
H4CB	0.6017	0.3710	0.9150	0.046*	
C5A	0.4596 (3)	0.3952 (2)	0.2307 (2)	0.0353 (7)	
H5A	0.5533	0.4012	0.2544	0.042*	
N1D	0.8612 (3)	0.11725 (18)	0.81367 (18)	0.0328 (6)	
H1D	0.9064	0.1151	0.7760	0.036 (9)*	
N1C	0.6842 (3)	0.36454 (18)	0.78397 (18)	0.0328 (6)	
H1CB	0.5978	0.3625	0.7648	0.034 (9)*	
N1B	0.4407 (3)	0.14033 (19)	0.27794 (17)	0.0326 (6)	
H1B	0.5267	0.1416	0.2946	0.034 (9)*	
N1A	0.2526 (3)	0.38643 (18)	0.25311 (18)	0.0320 (6)	
H1A	0.2073	0.3857	0.2904	0.050 (11)*	
C6A	0.3906 (3)	0.3959 (2)	0.2921 (2)	0.0312 (7)	
C5D	0.6560 (3)	0.1176 (2)	0.8379 (2)	0.0342 (7)	
H5D	0.5625	0.1154	0.8151	0.041*	
N5D	0.6668 (3)	0.1283 (2)	1.0000 (2)	0.0488 (8)	
H5DA	0.7134	0.1324	1.0606	0.059*	
H5DB	0.5805	0.1267	0.9804	0.059*	
N5C	1.0869 (3)	0.3771 (2)	0.8860 (2)	0.0480 (8)	
H5CA	1.1284	0.3813	0.9473	0.058*	
H5CB	1.1319	0.3760	0.8483	0.058*	
N6A	0.4492 (3)	0.4047 (2)	0.38832 (18)	0.0436 (7)	
H6AA	0.5357	0.4104	0.4151	0.052*	
H6AB	0.4004	0.4047	0.4234	0.052*	
O9	0.0270 (3)	0.0924 (2)	0.7016 (2)	0.0576 (7)	
C6C	0.7456 (3)	0.3616 (2)	0.7173 (2)	0.0314 (7)	
C5C	0.8827 (3)	0.3658 (2)	0.7500 (2)	0.0341 (7)	
H5C	0.9284	0.3642	0.7073	0.041*	
H9C	0.048 (4)	0.0364 (17)	0.687 (3)	0.051*	
C6D	0.7236 (3)	0.1144 (2)	0.7752 (2)	0.0306 (7)	
N6D	0.6658 (3)	0.10910 (19)	0.68015 (18)	0.0393 (7)	
H6DA	0.5797	0.1075	0.6538	0.047*	
H6DB	0.7146	0.1073	0.6449	0.047*	
N6C	0.6642 (3)	0.3551 (2)	0.62472 (19)	0.0445 (7)	
H6CA	0.6977	0.3533	0.5801	0.053*	
H6CB	0.5783	0.3528	0.6099	0.053*	
C6B	0.3847 (3)	0.1444 (2)	0.3472 (2)	0.0292 (7)	
N6B	0.4690 (3)	0.1497 (2)	0.43876 (19)	0.0444 (7)	
H6BA	0.5544	0.1503	0.4516	0.053*	
H6BB	0.4379	0.1524	0.4847	0.053*	
H9D	0.087 (3)	0.135 (2)	0.703 (3)	0.067*	
O10	0.3179 (3)	0.3727 (4)	0.5185 (2)	0.0917 (11)	
H10C	0.340 (6)	0.359 (5)	0.573 (2)	0.138*	
H10D	0.241 (3)	0.345 (4)	0.495 (4)	0.138*	
O11	0.0766 (4)	0.4674 (3)	0.5928 (2)	0.0791 (9)	
H11C	0.023 (5)	0.411 (3)	0.568 (4)	0.119*	
H11D	0.130 (5)	0.445 (4)	0.641 (3)	0.119*	
O12	0.7554 (3)	0.3892 (2)	0.14932 (19)	0.0575 (7)	
H12A	0.769 (5)	0.346 (3)	0.183 (3)	0.086*	
H12B	0.773 (5)	0.447 (2)	0.185 (3)	0.086*	
O13	0.7446 (3)	0.3873 (2)	0.4608 (2)	0.0624 (8)	
H13C	0.750 (5)	0.329 (2)	0.434 (3)	0.094*	
H13D	0.795 (4)	0.426 (3)	0.450 (4)	0.094*	
O14	0.0675 (3)	0.3836 (2)	0.3461 (2)	0.0635 (8)	
H14C	0.016 (4)	0.332 (2)	0.333 (4)	0.095*	
H14D	0.032 (5)	0.430 (3)	0.369 (3)	0.095*	
O15	0.7919 (3)	0.0860 (3)	0.5281 (2)	0.0716 (9)	
H15C	0.871 (3)	0.087 (4)	0.567 (3)	0.107*	
H15D	0.792 (5)	0.115 (4)	0.484 (3)	0.107*	
O16	0.3718 (3)	0.1016 (2)	0.5870 (2)	0.0604 (7)	
H16C	0.327 (4)	0.047 (2)	0.560 (3)	0.091*	
H16D	0.338 (4)	0.136 (3)	0.621 (3)	0.091*	
O17	0.3458 (3)	0.1177 (2)	0.90904 (19)	0.0591 (7)	
H17A	0.355 (5)	0.164 (3)	0.882 (3)	0.089*	
H17B	0.314 (4)	0.064 (2)	0.870 (3)	0.089*	
O18	1.086 (4)	0.250 (3)	0.5021 (19)	0.078 (8)	0.36 (7)
H18C	1.057 (17)	0.192 (7)	0.464 (11)	0.117*	0.36 (7)
H18D	1.138 (15)	0.220 (10)	0.542 (2)	0.117*	0.36 (7)
O18B	1.031 (4)	0.2505 (15)	0.5241 (19)	0.117 (8)	0.64 (7)
H18E	1.001 (13)	0.198 (7)	0.478 (7)	0.175*	0.64 (7)
H18F	1.096 (10)	0.227 (8)	0.561 (3)	0.175*	0.64 (7)

### Atomic displacement parameters (Å^2^)

**Table:**

	*U*^11^	*U*^22^	*U*^33^	*U*^12^	*U*^13^	*U*^23^
S1	0.0261 (4)	0.0418 (4)	0.0255 (4)	0.0006 (3)	0.0080 (3)	0.0074 (3)
O1	0.0445 (15)	0.0786 (18)	0.0312 (13)	0.0040 (13)	0.0073 (11)	0.0193 (12)
O2	0.0492 (16)	0.0530 (15)	0.0542 (16)	−0.0132 (12)	0.0171 (13)	−0.0020 (12)
C2C	0.0329 (17)	0.0253 (15)	0.0291 (16)	0.0034 (13)	0.0099 (14)	0.0072 (12)
O3	0.0605 (17)	0.0584 (16)	0.0480 (15)	0.0228 (13)	0.0249 (13)	0.0176 (12)
O4	0.0277 (13)	0.0814 (17)	0.0383 (14)	−0.0022 (12)	0.0110 (11)	0.0190 (12)
C4C	0.0313 (17)	0.0338 (16)	0.0319 (17)	0.0028 (13)	0.0128 (14)	0.0109 (13)
S2	0.0249 (4)	0.0470 (5)	0.0300 (4)	0.0049 (4)	0.0083 (3)	0.0099 (4)
O5	0.066 (2)	0.076 (2)	0.121 (3)	−0.0015 (18)	0.010 (2)	0.058 (2)
O6	0.0360 (14)	0.112 (3)	0.0366 (14)	0.0129 (15)	0.0156 (12)	0.0096 (15)
O7	0.0368 (15)	0.0607 (19)	0.0434 (15)	0.0175 (14)	0.0090 (12)	0.0106 (14)
O8	0.0495 (18)	0.083 (2)	0.0342 (16)	0.0150 (16)	−0.0005 (14)	−0.0084 (14)
S2B	0.0249 (4)	0.0470 (5)	0.0300 (4)	0.0049 (4)	0.0083 (3)	0.0099 (4)
O5B	0.066 (2)	0.076 (2)	0.121 (3)	−0.0015 (18)	0.010 (2)	0.058 (2)
O6B	0.0360 (14)	0.112 (3)	0.0366 (14)	0.0129 (15)	0.0156 (12)	0.0096 (15)
O7B	0.0368 (15)	0.0607 (19)	0.0434 (15)	0.0175 (14)	0.0090 (12)	0.0106 (14)
O8B	0.0495 (18)	0.083 (2)	0.0342 (16)	0.0150 (16)	−0.0005 (14)	−0.0084 (14)
N3B	0.0254 (14)	0.0342 (14)	0.0264 (13)	0.0032 (11)	0.0085 (11)	0.0059 (11)
C2B	0.0278 (16)	0.0286 (15)	0.0258 (15)	0.0013 (12)	0.0086 (13)	0.0047 (12)
C2A	0.0333 (18)	0.0265 (15)	0.0300 (17)	0.0010 (13)	0.0092 (14)	0.0065 (12)
N3A	0.0299 (15)	0.0394 (15)	0.0262 (14)	0.0021 (12)	0.0066 (12)	0.0081 (11)
C2D	0.0339 (18)	0.0295 (16)	0.0289 (17)	0.0006 (13)	0.0062 (14)	0.0055 (13)
N4B	0.0296 (15)	0.0653 (19)	0.0290 (14)	0.0063 (13)	0.0115 (12)	0.0132 (13)
C4B	0.0270 (16)	0.0278 (15)	0.0323 (17)	0.0035 (12)	0.0107 (14)	0.0064 (13)
N4A	0.0290 (15)	0.0587 (18)	0.0313 (15)	0.0011 (13)	0.0086 (12)	0.0092 (13)
N3D	0.0366 (15)	0.0377 (14)	0.0267 (14)	0.0009 (12)	0.0089 (12)	0.0078 (11)
N3C	0.0268 (14)	0.0371 (14)	0.0276 (13)	0.0025 (11)	0.0079 (11)	0.0088 (11)
C4A	0.0328 (18)	0.0368 (17)	0.0315 (17)	0.0076 (14)	0.0117 (15)	0.0082 (14)
N5B	0.0251 (15)	0.0682 (19)	0.0378 (16)	0.0084 (13)	0.0135 (13)	0.0151 (14)
C5B	0.0281 (17)	0.0400 (17)	0.0302 (17)	0.0011 (14)	0.0130 (14)	0.0063 (13)
N5A	0.0343 (16)	0.083 (2)	0.0296 (15)	0.0075 (15)	0.0110 (13)	0.0155 (15)
C4D	0.0343 (18)	0.0340 (17)	0.0318 (17)	0.0015 (14)	0.0111 (15)	0.0095 (13)
N4D	0.0305 (16)	0.072 (2)	0.0323 (15)	0.0026 (14)	0.0081 (13)	0.0125 (14)
N4C	0.0272 (14)	0.0567 (17)	0.0313 (15)	0.0043 (13)	0.0108 (12)	0.0120 (13)
C5A	0.0278 (17)	0.0456 (19)	0.0311 (17)	0.0024 (14)	0.0081 (14)	0.0103 (14)
N1D	0.0328 (15)	0.0397 (15)	0.0274 (14)	0.0041 (12)	0.0126 (12)	0.0077 (11)
N1C	0.0250 (15)	0.0385 (15)	0.0326 (15)	0.0026 (11)	0.0068 (12)	0.0097 (11)
N1B	0.0224 (14)	0.0432 (15)	0.0297 (14)	0.0029 (11)	0.0078 (11)	0.0055 (11)
N1A	0.0342 (15)	0.0360 (14)	0.0265 (13)	0.0005 (11)	0.0123 (12)	0.0069 (11)
C6A	0.0349 (18)	0.0291 (16)	0.0254 (16)	0.0030 (13)	0.0070 (14)	0.0043 (12)
C5D	0.0318 (17)	0.0385 (17)	0.0312 (17)	0.0036 (14)	0.0084 (14)	0.0108 (14)
N5D	0.0394 (17)	0.078 (2)	0.0328 (16)	0.0070 (16)	0.0145 (14)	0.0170 (15)
N5C	0.0271 (15)	0.087 (2)	0.0361 (16)	0.0097 (15)	0.0136 (13)	0.0226 (15)
N6A	0.0393 (16)	0.0636 (19)	0.0244 (14)	0.0059 (14)	0.0079 (13)	0.0080 (13)
O9	0.0556 (18)	0.0478 (16)	0.082 (2)	0.0062 (14)	0.0410 (16)	0.0139 (16)
C6C	0.0383 (19)	0.0264 (15)	0.0272 (16)	0.0030 (14)	0.0093 (14)	0.0054 (12)
C5C	0.0335 (18)	0.0437 (18)	0.0284 (17)	0.0058 (14)	0.0140 (14)	0.0104 (14)
C6D	0.0349 (18)	0.0231 (15)	0.0301 (16)	0.0026 (13)	0.0078 (14)	0.0054 (12)
N6D	0.0400 (16)	0.0504 (17)	0.0263 (14)	0.0070 (13)	0.0088 (13)	0.0113 (12)
N6C	0.0390 (17)	0.0605 (18)	0.0306 (15)	0.0060 (14)	0.0082 (13)	0.0107 (13)
C6B	0.0315 (17)	0.0306 (16)	0.0239 (15)	0.0032 (13)	0.0081 (13)	0.0058 (12)
N6B	0.0312 (15)	0.072 (2)	0.0280 (15)	0.0078 (14)	0.0077 (13)	0.0110 (14)
O10	0.063 (2)	0.162 (3)	0.057 (2)	−0.007 (2)	0.0246 (18)	0.038 (2)
O11	0.086 (3)	0.085 (2)	0.058 (2)	0.0325 (19)	0.0139 (18)	0.0153 (18)
O12	0.0738 (19)	0.0545 (17)	0.0404 (16)	0.0049 (16)	0.0163 (15)	0.0102 (12)
O13	0.065 (2)	0.0629 (19)	0.0613 (19)	0.0083 (16)	0.0290 (16)	0.0058 (15)
O14	0.0565 (19)	0.0651 (19)	0.081 (2)	0.0056 (15)	0.0421 (17)	0.0123 (17)
O15	0.0555 (18)	0.104 (2)	0.060 (2)	−0.0010 (18)	0.0215 (16)	0.0299 (18)
O16	0.0509 (18)	0.0678 (19)	0.0597 (19)	0.0098 (14)	0.0239 (15)	−0.0009 (15)
O17	0.085 (2)	0.0526 (17)	0.0372 (15)	−0.0002 (16)	0.0191 (15)	0.0127 (12)
O18	0.077 (13)	0.081 (10)	0.052 (9)	−0.021 (8)	−0.009 (8)	0.025 (7)
O18B	0.117 (14)	0.095 (6)	0.077 (8)	0.010 (8)	−0.028 (9)	−0.002 (5)

### Geometric parameters (Å, º)

**Table:**

S1—O1	1.459 (2)	C5A—H5A	0.9300
S1—O3	1.461 (2)	N1D—C6D	1.371 (4)
S1—O4	1.469 (2)	N1D—H1D	0.8600
S1—O2	1.471 (2)	N1C—C6C	1.372 (4)
C2C—N3C	1.319 (4)	N1C—H1CB	0.8600
C2C—N4C	1.327 (4)	N1B—C6B	1.363 (4)
C2C—N1C	1.362 (4)	N1B—H1B	0.8600
C4C—N5C	1.330 (4)	N1A—C6A	1.368 (4)
C4C—N3C	1.361 (4)	N1A—H1A	0.8600
C4C—C5C	1.399 (4)	C6A—N6A	1.337 (4)
S2—O8	1.449 (4)	C5D—C6D	1.368 (4)
S2—O5	1.450 (4)	C5D—H5D	0.9300
S2—O6	1.453 (3)	N5D—H5DA	0.8600
S2—O7	1.471 (3)	N5D—H5DB	0.8600
S2B—O8B	1.447 (18)	N5C—H5CA	0.8600
S2B—O5B	1.449 (19)	N5C—H5CB	0.8600
S2B—O6B	1.452 (18)	N6A—H6AA	0.8600
S2B—O7B	1.469 (18)	N6A—H6AB	0.8600
N3B—C2B	1.315 (4)	O9—H9C	0.797 (18)
N3B—C4B	1.354 (4)	O9—H9D	0.829 (19)
C2B—N4B	1.326 (4)	C6C—N6C	1.339 (4)
C2B—N1B	1.360 (4)	C6C—C5C	1.364 (4)
C2A—N3A	1.314 (4)	C5C—H5C	0.9300
C2A—N4A	1.326 (4)	C6D—N6D	1.327 (4)
C2A—N1A	1.362 (4)	N6D—H6DA	0.8600
N3A—C4A	1.356 (4)	N6D—H6DB	0.8600
C2D—N3D	1.319 (4)	N6C—H6CA	0.8600
C2D—N4D	1.329 (4)	N6C—H6CB	0.8600
C2D—N1D	1.366 (4)	C6B—N6B	1.339 (4)
N4B—H4BA	0.8600	N6B—H6BA	0.8600
N4B—H4BB	0.8600	N6B—H6BB	0.8600
C4B—N5B	1.330 (4)	O10—H10C	0.826 (19)
C4B—C5B	1.400 (4)	O10—H10D	0.81 (2)
N4A—H4AA	0.8600	O11—H11C	0.856 (19)
N4A—H4AB	0.8600	O11—H11D	0.854 (19)
N3D—C4D	1.353 (4)	O12—H12A	0.821 (19)
C4A—N5A	1.332 (4)	O12—H12B	0.819 (19)
C4A—C5A	1.404 (4)	O13—H13C	0.821 (19)
N5B—H5BA	0.8600	O13—H13D	0.808 (19)
N5B—H5BB	0.8600	O14—H14C	0.807 (19)
C5B—C6B	1.371 (4)	O14—H14D	0.813 (19)
C5B—H5B	0.9300	O15—H15C	0.836 (19)
N5A—H5AA	0.8600	O15—H15D	0.830 (19)
N5A—H5AB	0.8600	O16—H16C	0.808 (19)
C4D—N5D	1.341 (4)	O16—H16D	0.816 (19)
C4D—C5D	1.384 (4)	O17—H17A	0.820 (19)
N4D—H4DA	0.8600	O17—H17B	0.807 (19)
N4D—H4DB	0.8600	O18—H18C	0.84 (2)
N4C—H4CA	0.8600	O18—H18D	0.84 (2)
N4C—H4CB	0.8600	O18B—H18E	0.84 (2)
C5A—C6A	1.366 (4)	O18B—H18F	0.83 (2)
			
O1—S1—O3	110.02 (14)	C2C—N4C—H4CB	120.0
O1—S1—O4	110.02 (14)	H4CA—N4C—H4CB	120.0
O3—S1—O4	109.89 (15)	C6A—C5A—C4A	118.4 (3)
O1—S1—O2	109.83 (15)	C6A—C5A—H5A	120.8
O3—S1—O2	108.84 (15)	C4A—C5A—H5A	120.8
O4—S1—O2	108.21 (14)	C2D—N1D—C6D	120.9 (3)
N3C—C2C—N4C	121.1 (3)	C2D—N1D—H1D	119.6
N3C—C2C—N1C	122.5 (3)	C6D—N1D—H1D	119.6
N4C—C2C—N1C	116.4 (3)	C2C—N1C—C6C	121.2 (3)
N5C—C4C—N3C	116.1 (3)	C2C—N1C—H1CB	119.4
N5C—C4C—C5C	121.3 (3)	C6C—N1C—H1CB	119.4
N3C—C4C—C5C	122.6 (3)	C2B—N1B—C6B	121.1 (3)
O8—S2—O5	109.5 (3)	C2B—N1B—H1B	119.4
O8—S2—O6	110.5 (3)	C6B—N1B—H1B	119.4
O5—S2—O6	108.3 (3)	C2A—N1A—C6A	120.7 (3)
O8—S2—O7	110.7 (2)	C2A—N1A—H1A	119.7
O5—S2—O7	108.3 (2)	C6A—N1A—H1A	119.7
O6—S2—O7	109.5 (3)	N6A—C6A—C5A	124.1 (3)
O8B—S2B—O5B	109 (2)	N6A—C6A—N1A	117.6 (3)
O8B—S2B—O6B	110 (3)	C5A—C6A—N1A	118.3 (3)
O5B—S2B—O6B	109 (3)	C6D—C5D—C4D	119.0 (3)
O8B—S2B—O7B	110 (2)	C6D—C5D—H5D	120.5
O5B—S2B—O7B	108 (2)	C4D—C5D—H5D	120.5
O6B—S2B—O7B	110 (3)	C4D—N5D—H5DA	120.0
C2B—N3B—C4B	117.1 (3)	C4D—N5D—H5DB	120.0
N3B—C2B—N4B	121.0 (3)	H5DA—N5D—H5DB	120.0
N3B—C2B—N1B	122.8 (3)	C4C—N5C—H5CA	120.0
N4B—C2B—N1B	116.2 (3)	C4C—N5C—H5CB	120.0
N3A—C2A—N4A	119.7 (3)	H5CA—N5C—H5CB	120.0
N3A—C2A—N1A	123.1 (3)	C6A—N6A—H6AA	120.0
N4A—C2A—N1A	117.2 (3)	C6A—N6A—H6AB	120.0
C2A—N3A—C4A	117.3 (3)	H6AA—N6A—H6AB	120.0
N3D—C2D—N4D	120.0 (3)	H9C—O9—H9D	107 (4)
N3D—C2D—N1D	122.9 (3)	N6C—C6C—C5C	125.6 (3)
N4D—C2D—N1D	117.0 (3)	N6C—C6C—N1C	116.4 (3)
C2B—N4B—H4BA	120.0	C5C—C6C—N1C	118.0 (3)
C2B—N4B—H4BB	120.0	C6C—C5C—C4C	118.4 (3)
H4BA—N4B—H4BB	120.0	C6C—C5C—H5C	120.8
N5B—C4B—N3B	116.1 (3)	C4C—C5C—H5C	120.8
N5B—C4B—C5B	121.0 (3)	N6D—C6D—C5D	124.9 (3)
N3B—C4B—C5B	122.9 (3)	N6D—C6D—N1D	117.7 (3)
C2A—N4A—H4AA	120.0	C5D—C6D—N1D	117.4 (3)
C2A—N4A—H4AB	120.0	C6D—N6D—H6DA	120.0
H4AA—N4A—H4AB	120.0	C6D—N6D—H6DB	120.0
C2D—N3D—C4D	116.7 (3)	H6DA—N6D—H6DB	120.0
C2C—N3C—C4C	117.3 (3)	C6C—N6C—H6CA	120.0
N5A—C4A—N3A	116.7 (3)	C6C—N6C—H6CB	120.0
N5A—C4A—C5A	120.9 (3)	H6CA—N6C—H6CB	120.0
N3A—C4A—C5A	122.3 (3)	N6B—C6B—N1B	117.0 (3)
C4B—N5B—H5BA	120.0	N6B—C6B—C5B	124.8 (3)
C4B—N5B—H5BB	120.0	N1B—C6B—C5B	118.2 (3)
H5BA—N5B—H5BB	120.0	C6B—N6B—H6BA	120.0
C6B—C5B—C4B	117.9 (3)	C6B—N6B—H6BB	120.0
C6B—C5B—H5B	121.1	H6BA—N6B—H6BB	120.0
C4B—C5B—H5B	121.1	H10C—O10—H10D	99 (6)
C4A—N5A—H5AA	120.0	H11C—O11—H11D	96 (5)
C4A—N5A—H5AB	120.0	H12A—O12—H12B	109 (4)
H5AA—N5A—H5AB	120.0	H13C—O13—H13D	106 (5)
N5D—C4D—N3D	115.9 (3)	H14C—O14—H14D	105 (5)
N5D—C4D—C5D	121.0 (3)	H15C—O15—H15D	110 (5)
N3D—C4D—C5D	123.1 (3)	H16C—O16—H16D	110 (5)
C2D—N4D—H4DA	120.0	H17A—O17—H17B	110 (5)
C2D—N4D—H4DB	120.0	H18C—O18—H18D	88 (10)
H4DA—N4D—H4DB	120.0	H18E—O18B—H18F	99 (10)
C2C—N4C—H4CA	120.0		
			
C4B—N3B—C2B—N4B	179.2 (3)	N3B—C2B—N1B—C6B	0.9 (4)
C4B—N3B—C2B—N1B	−1.1 (4)	N4B—C2B—N1B—C6B	−179.4 (3)
N4A—C2A—N3A—C4A	179.5 (3)	N3A—C2A—N1A—C6A	1.0 (4)
N1A—C2A—N3A—C4A	−0.2 (4)	N4A—C2A—N1A—C6A	−178.8 (3)
C2B—N3B—C4B—N5B	−179.0 (3)	C4A—C5A—C6A—N6A	179.8 (3)
C2B—N3B—C4B—C5B	0.5 (4)	C4A—C5A—C6A—N1A	0.3 (4)
N4D—C2D—N3D—C4D	179.8 (3)	C2A—N1A—C6A—N6A	179.4 (3)
N1D—C2D—N3D—C4D	0.1 (4)	C2A—N1A—C6A—C5A	−1.0 (4)
N4C—C2C—N3C—C4C	−179.2 (3)	N5D—C4D—C5D—C6D	179.6 (3)
N1C—C2C—N3C—C4C	0.1 (4)	N3D—C4D—C5D—C6D	−0.4 (5)
N5C—C4C—N3C—C2C	179.6 (3)	C2C—N1C—C6C—N6C	179.8 (3)
C5C—C4C—N3C—C2C	−0.3 (4)	C2C—N1C—C6C—C5C	−0.5 (4)
C2A—N3A—C4A—N5A	178.8 (3)	N6C—C6C—C5C—C4C	180.0 (3)
C2A—N3A—C4A—C5A	−0.5 (4)	N1C—C6C—C5C—C4C	0.2 (4)
N5B—C4B—C5B—C6B	179.8 (3)	N5C—C4C—C5C—C6C	−179.8 (3)
N3B—C4B—C5B—C6B	0.3 (4)	N3C—C4C—C5C—C6C	0.2 (5)
C2D—N3D—C4D—N5D	−179.9 (3)	C4D—C5D—C6D—N6D	−179.3 (3)
C2D—N3D—C4D—C5D	0.1 (4)	C4D—C5D—C6D—N1D	0.4 (4)
N5A—C4A—C5A—C6A	−178.8 (3)	C2D—N1D—C6D—N6D	179.5 (3)
N3A—C4A—C5A—C6A	0.5 (5)	C2D—N1D—C6D—C5D	−0.2 (4)
N3D—C2D—N1D—C6D	0.0 (4)	C2B—N1B—C6B—N6B	179.6 (3)
N4D—C2D—N1D—C6D	−179.7 (3)	C2B—N1B—C6B—C5B	0.0 (4)
N3C—C2C—N1C—C6C	0.3 (4)	C4B—C5B—C6B—N6B	179.8 (3)
N4C—C2C—N1C—C6C	179.6 (3)	C4B—C5B—C6B—N1B	−0.6 (4)

### Hydrogen-bond geometry (Å, º)

**Table:**

*D*—H···*A*	*D*—H	H···*A*	*D*···*A*	*D*—H···*A*
N4*B*—H4*BA*···O17^i^	0.86	2.15	2.967 (4)	159
N4*B*—H4*BB*···O8	0.86	2.07	2.905 (4)	162
N4*B*—H4*BB*···O5*B*	0.86	2.38	3.19 (4)	157
N4*A*—H4*AA*···N3*C*^ii^	0.86	2.16	3.017 (4)	172
N4*A*—H4*AB*···O14	0.86	2.56	3.262 (4)	139
N5*B*—H5*BA*···N3*D*^ii^	0.86	2.22	3.074 (4)	174
N5*B*—H5*BB*···O7^iii^	0.86	2.20	3.040 (4)	166
N5*B*—H5*BB*···O7*B*^iii^	0.86	2.10	2.93 (3)	163
N5*A*—H5*AA*···O1^i^	0.86	2.25	2.993 (3)	145
N5*A*—H5*AB*···O12	0.86	2.21	3.066 (4)	174
N4*D*—H4*DA*···N3*B*^iv^	0.86	2.16	3.013 (4)	176
N4*D*—H4*DB*···O17^v^	0.86	2.57	3.248 (4)	137
N4*C*—H4*CA*···O12^vi^	0.86	2.14	2.953 (4)	158
N4*C*—H4*CB*···O1	0.86	2.16	2.983 (3)	160
N1*D*—H1*D*···O9^v^	0.86	1.97	2.826 (4)	170
N1*C*—H1*CB*···O4	0.86	1.86	2.709 (3)	172
N1*B*—H1*B*···O6	0.86	1.92	2.757 (6)	166
N1*B*—H1*B*···O6*B*	0.86	1.91	2.75 (7)	164
N1*A*—H1*A*···O14	0.86	1.95	2.793 (4)	166
N5*D*—H5*DA*···O8^vi^	0.86	2.31	3.046 (4)	143
N5*D*—H5*DA*···O5*B*^vi^	0.86	2.23	2.99 (5)	147
N5*D*—H5*DB*···O17	0.86	2.34	3.193 (4)	173
N5*C*—H5*CA*···N3*A*^iv^	0.86	2.14	2.990 (4)	172
N5*C*—H5*CB*···O3^v^	0.86	2.06	2.905 (3)	169
N6*A*—H6*AA*···O13	0.86	2.14	2.981 (4)	166
N6*A*—H6*AB*···O10	0.86	2.02	2.855 (4)	164
O9—H9*C*···O7^vii^	0.80 (2)	1.98 (2)	2.770 (4)	171 (4)
O9—H9*C*···O8*B*^vii^	0.80 (2)	2.24 (5)	2.84 (3)	133 (3)
N6*D*—H6*DA*···O16	0.86	2.08	2.937 (4)	179
N6*D*—H6*DB*···O15	0.86	2.16	3.005 (4)	166
N6*C*—H6*CA*···O13	0.86	2.14	2.970 (4)	162
N6*B*—H6*BA*···O6	0.86	2.60	3.284 (5)	137
N6*B*—H6*BA*···O6*B*	0.86	2.64	3.31 (4)	135
N6*B*—H6*BA*···O15	0.86	2.63	3.427 (4)	154
N6*B*—H6*BB*···O16	0.86	2.10	2.923 (4)	159
O9—H9*D*···O2	0.83 (2)	1.93 (2)	2.759 (4)	173 (4)
O10—H10*C*···O4	0.83 (2)	1.96 (2)	2.780 (4)	172 (6)
O10—H10*D*···O14	0.81 (2)	2.51 (6)	3.026 (5)	123 (6)
O10—H10*D*···O18^iii^	0.81 (2)	2.09 (5)	2.82 (3)	151 (6)
O11—H11*C*···O18^iii^	0.86 (2)	2.40 (7)	2.97 (4)	124 (5)
O11—H11*C*···O18*B*^iii^	0.86 (2)	2.12 (4)	2.826 (18)	139 (5)
O11—H11*D*···O3	0.85 (2)	1.90 (2)	2.745 (4)	171 (5)
O12—H12*A*···O5	0.82 (2)	1.98 (2)	2.795 (4)	172 (5)
O12—H12*A*···O5*B*	0.82 (2)	2.05 (4)	2.81 (3)	156 (5)
O12—H12*B*···O3^viii^	0.82 (2)	2.06 (2)	2.856 (4)	165 (5)
O13—H13*C*···O6	0.82 (2)	2.15 (2)	2.956 (6)	167 (5)
O13—H13*C*···O6*B*	0.82 (2)	2.06 (7)	2.87 (6)	167 (5)
O13—H13*D*···O11^viii^	0.81 (2)	2.26 (2)	3.063 (5)	175 (5)
O14—H14*C*···O5^iii^	0.81 (2)	2.06 (3)	2.792 (4)	152 (5)
O14—H14*C*···O7*B*^iii^	0.81 (2)	2.21 (4)	2.98 (4)	159 (5)
O14—H14*D*···O11^ix^	0.81 (2)	1.95 (2)	2.761 (4)	172 (5)
O15—H15*C*···O9^v^	0.84 (2)	2.10 (3)	2.894 (4)	158 (5)
O15—H15*D*···O6	0.83 (2)	2.02 (3)	2.808 (7)	158 (5)
O15—H15*D*···O6*B*	0.83 (2)	2.11 (8)	2.90 (8)	159 (5)
O16—H16*C*···O15^vii^	0.81 (2)	2.05 (2)	2.855 (5)	171 (5)
O16—H16*D*···O2	0.82 (2)	2.07 (2)	2.882 (4)	175 (5)
O17—H17*A*···O1	0.82 (2)	2.09 (2)	2.878 (4)	162 (4)
O17—H17*B*···O8^vii^	0.81 (2)	2.07 (2)	2.837 (4)	160 (5)
O17—H17*B*···O8*B*^vii^	0.81 (2)	1.96 (4)	2.74 (3)	163 (5)
O18—H18*C*···O7	0.84 (2)	2.04 (11)	2.78 (2)	147 (18)
O18—H18*D*···O2^v^	0.84 (2)	2.21 (2)	2.92 (2)	143 (5)
O18*B*—H18*E*···O7*B*	0.84 (2)	1.83 (11)	2.49 (4)	134 (13)
O18*B*—H18*F*···O2^v^	0.83 (2)	2.10 (2)	2.857 (14)	152 (6)

Symmetry codes: (i) *x*, *y*, *z*−1; (ii) *x*−1, *y*, *z*−1; (iii) *x*−1, *y*, *z*; (iv) *x*+1, *y*, *z*+1; (v) *x*+1, *y*, *z*; (vi) *x*, *y*, *z*+1; (vii) −*x*+1, −*y*, −*z*+1; (viii) −*x*+1, −*y*+1, −*z*+1; (ix) −*x*, −*y*+1, −*z*+1.
